# Brain hemodynamic response in Examiner–Examinee dyads during spatial short-term memory task: an fNIRS study

**DOI:** 10.1007/s00221-021-06073-0

**Published:** 2021-03-22

**Authors:** Francesco Panico, Stefania De Marco, Laura Sagliano, Francesca D’Olimpio, Dario Grossi, Luigi Trojano

**Affiliations:** Department of Psychology, University of Campania “Luigi Vanvitelli”, Viale Ellittico 31, 81100 Caserta, Italy

**Keywords:** Corsi test, fNIRS, Working Memory, Workload, Joint actions

## Abstract

The Corsi Block-Tapping test (CBT) is a measure of spatial working memory (WM) in clinical practice, requiring an examinee to reproduce sequences of cubes tapped by an examiner. CBT implies complementary behaviors in the examiners and the examinees, as they have to attend a precise turn taking. Previous studies demonstrated that the Prefrontal Cortex (PFC) is activated during CBT, but scarce evidence is available on the neural correlates of CBT in the real setting. We assessed PFC activity in dyads of examiner–examinee participants while completing the real version of CBT, during conditions of increasing and exceeding workload. This procedure allowed to investigate whether brain activity in the dyads is coordinated. Results in the examinees showed that PFC activity was higher when the workload approached or reached participants’ spatial WM span, and lower during workload conditions that were largely below or above their span. Interestingly, findings in the examiners paralleled the ones in the examinees, as examiners’ brain activity increased and decreased in a similar way as the examinees’ one. In the examiners, higher left-hemisphere activity was observed suggesting the likely activation of non-spatial WM processes. Data support a bell-shaped relationship between cognitive load and brain activity, and provide original insights on the cognitive processes activated in the examiner during CBT.

**Supplementary Information:**

The online version contains supplementary material available at 10.1007/s00221-021-06073-0.

## Introduction

Working Memory (WM; Baddeley 2003) is the ability to hold verbal and spatial information for keeping and manipulating relevant task-related material to complete an action (Chai et al. 2018). To date the Corsi Block-Tapping test (CBT; Corsi 1973) is one of the most commonly used measures of spatial WM in clinical practice. The task requires the proband to reproduce a series of cubes mimicking the sequence tapped by the examiner. By presenting sequences of increasing lengths, it is possible to assess the short-term visuospatial span.

A few studies attempted at elucidating the neural correlates of WM as measured by the CBT (Bor et al. 2006; Toepper et al. 2010). A Positron Emission Tomography (PET) study (Bor et al. 2006) showed that in healthy individuals CBT activated the prefrontal cortex (PFC), and that patients with large frontal lesions were significantly impaired on this task, particularly when the right dorsolateral PFC was damaged. Later, a functional Magnetic Resonance Imaging study (fMRI) on healthy volunteers (Toepper et al. 2010) confirmed the involvement of PFC during CBT execution. More recently, Lancia et al. (2018) investigated the neural correlates of CBT by means of functional Near-Infrared Spectroscopy (fNIRS), a non-invasive neuroimaging method assessing changes in brain hemodynamics (Scholkmann et al. 2014b; Pinti et al. 2018b). As compared to the other neuroimaging techniques, the fNIRS has greater temporal resolution, lower acquisition cost, less movement-related artifacts and greater flexibility when used to study individuals in an ecological environment during natural interactions, although at the cost of lower spatial resolution. In their study, Lancia et al. (2018) specifically assessed activation of the ventrolateral and dorsolateral PFC during a computerized version of CBT and a block-suppression computerized version of the CBT, which required inhibiting a response toward distractor cubes. The results suggested that both the ventrolateral and dorsolateral PFC were involved in both tasks, without region-specific activation patterns.

The above mentioned studies adopted computerized versions of the CBT (Bor et al. 2006; Toepper et al. 2010; Lancia et al. 2018), and some of them performed the task in the artificial environment of the fMRI/PET scanner (Bor et al. 2006; Toepper et al. 2010). As recent studies highlighted similarities and dissimilarities between standard and computerized CBT versions (Claessen et al. 2015; Robinson and Brewer 2016), in the present experiment we investigated prefrontal hemodynamic response in an ecological environment reproducing the actual clinical setting. In this perspective, only one study investigated neural correlates of CBT by fNIRS in highly realistic settings to support diagnosis of Alzheimer’s disease but no significant difference in prefrontal cortex activation was observed between patients and healthy participants (Perpetuini et al. 2019).

In our purpose, it would be worth considering that in a real setting the CBT activates complementary behaviors in the examiners and the examinees, who have to attend a precise turn taking, in which they alternatively observe and produce the cube sequences. While the examinee's brain activity was investigated before, no study has targeted brain activity patterns in the examiner. From hyperscanning studies, we can infer that this complementary performance could led to a coordination in neural activity, i.e., that the examiner's brain activity might mirror the examinee's one. For instance, Liu et al. (2016) showed increased inter-brain neural synchronization in PFC during a naturally occurring cooperative task involving face-to-face communication. Inter-brain synchrony was observed when participants performed the task together but not during an individual condition (Cui et al. 2012; Dommer et al. 2012; Jiang et al. 2012; Liu et al. 2015; Baker et al. 2016; Nguyen et al. 2020). In the real CBT administration, mimicking the sequences produced by another person can be considered as a form of imitation of movements directed toward spatial positions, and some findings support the hypothesis that action perception shares the same mechanisms as action performance (Meltzoff and Decety 2003; Kokal et al. 2009). Yet to date no study assessed the ongoing patterns of brain activation in the examiner during CBT administration. To fill this gap and to gather relevant information about possible brain synchronization during spatial WM tasks, in our experimental setup we monitored PFC activity in the dyads of Examiner–Examinee participants while completing the CBT.

In line with previous neuroimaging studies (Bor et al. 2006; Toepper et al. 2010), we expected that CBT execution would induce PFC activity and that this activity would increase as a function of the workload in the Examinees. Moreover, in line with previous findings on neural synchrony (Cui et al. 2012; Dommer et al. 2012; Jiang et al. 2012; Liu et al. 2015, 2016; Baker et al. 2016; Nguyen et al. 2020), and on shared common codes between action and perception (Meltzoff and Decety 2003; Kokal et al. 2009) we also speculated that the Examinees’ and Examiners’ brain activation in the dyads would parallel each other during the task.

## Materials and method

### Participants and experimental design

Sixty right-handed university students (31 female; average age = 22.48, SD = 2.34) voluntarily participated to this study. Participants had normal or corrected-to-normal vision, were naïve to the purposes of the study and were included only if they had not previously administered or completed a CBT examination.

Participants were informed that the aim of the experiment was to evaluate the contribution of different brain regions during completion of a neuropsychological test in a condition of interaction, by means of a non-invasive neuroimaging technique (fNIRS). The participants gave their written informed consent to take part in the experiment.

Participants were randomly divided in 2 groups: 30 participants were assigned to the role of Examiner (17 female; average age = 22.8, SD = 2.59), while 30 participants were assigned to the role of Examinee (14 female; average age = 22.17, SD = 2.05). Each experimental session involved two participants, one from the Examiner group and one from the Examinee group, who constituted a dyad.

The procedure was in agreement with 1975 Helsinki Declaration and was approved by the Local Ethic Committee.

### Corsi Block-Tapping test

The Italian standardized version of the Corsi Block-Tapping test (Spinnler and Tognoni 1987) was employed. The test material is made of nine cubes (3 × 3 × 3 cm) positioned in irregular order on a wooden board (23 × 28 cm). According to standardized instructions (Spinnler and Tognoni 1987), the Examinee was required to reproduce the same sequence of cubes (in the same order) tapped by the Examiner. The test started with sequences of two cubes, and series length gradually increased up to 10 units; for each series length three sequences were presented. The maximum series length for which the Examinee achieved two correct reproductions was considered as his/her spatial short-term memory span (range 2–10). To obtain a measure of brain activity in condition of increased workload, the administration of the CBT was extended beyond the Examinee’s span; so, all participants completed all the span sequences, independently of their span. This represented the only change with respect to the standard administration procedure in clinical practice.

Therefore, both the Examiner and the Examinee had to tap cubes organized in increasing sequences, but their instructions differed. The Examinee had to reproduce the observed sequences mimicking the Examiner; the Examiner had to read and rehearse the sequence of cubes to be tapped (the side of the cubes facing the Examiner are numbered), execute the sequence and then observe and record the cubes (i.e., the corresponding cube numbers) tapped by the Examinee. The two participants of each dyad acted in a complementary way: when the Examiner tapped the cubes, the Examinee had to observe him/her (phase 1 of the trial); when the Examinee tapped the cubes, the Examiner had to observe him/her and record the responses (phase 2 of the trial).

### functional Near-Infrared Spectroscopy (fNIRS)

Two 2 × 4-channel continuous wave fNIRS systems (OctaMon, Artinis Medical Systems, The Netherlands) were employed to record levels in oxygenated (O2Hb) and deoxygenated hemoglobin (HHb) over the bilateral PFC of the Examiner and the Examinee in a dyad. These devices measure the variations in light attenuation at two wavelengths, 758 and 840 nm. The O2Hb and HHb concentration levels (expressed in ΔμM), obtained using the modified Beer–Lambert law were displayed in real time. Data were acquired using the OxySoft software (OxySoft, Artinis Medical Systems, The Netherlands) at a frequency of 10 Hz. The differential pathlength factor (DPF) was selected individually for each participant according to his/her age (Duncan et al. 1996).

**Fig. 1:**
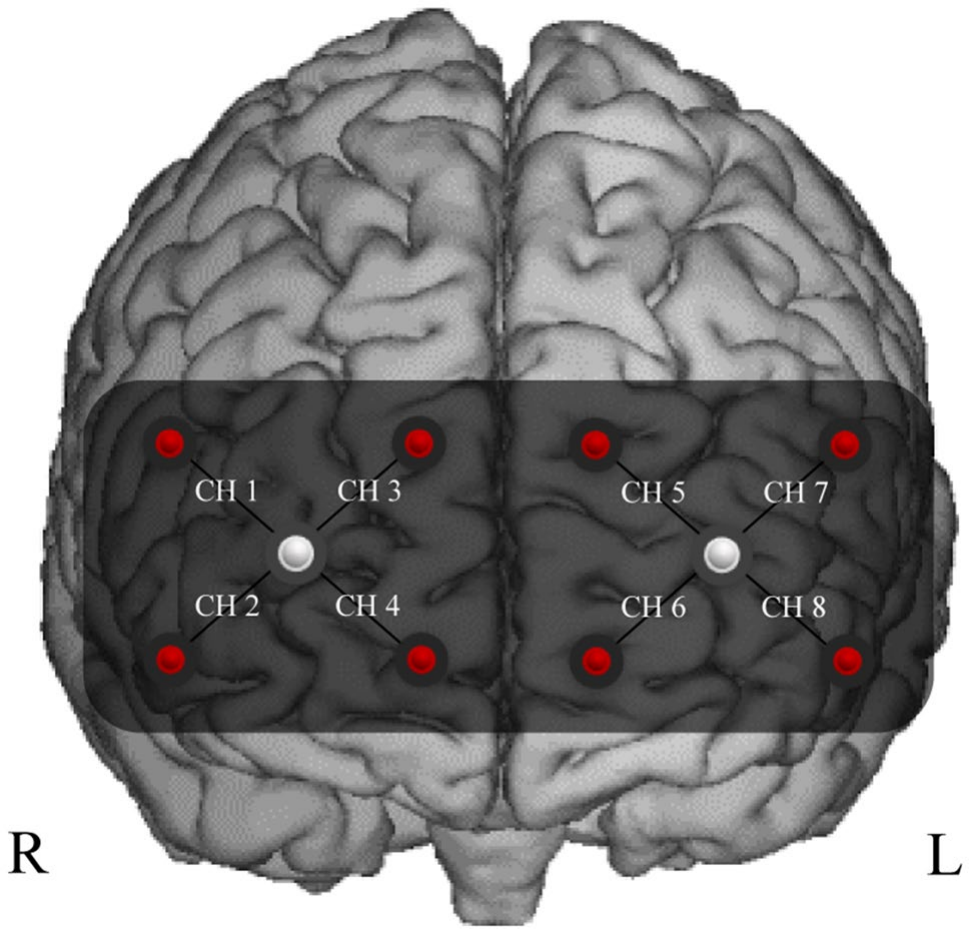
fNIRS headband. Location of the optodes on participant’s forehead with a flexible fNIRS sensor pad labeled from channel 1 to channel 8 (Ch 1–Ch 8). The headband included two receivers (in the center) and eight transmitters (in the periphery). Receivers were positioned on the line of FP1 and FP2 according to the international 10–20 system for the electroencephalography electrode placement. R and L indicate the right and left hemispheres, respectively

Eight LEDs bundles (four for each hemisphere) were utilized to carry out the light to the left and the right PFC, whereas two photodiodes (one for each hemispheres) with proprietary ambient light protection were used to collect the light emerging from the same cortical areas. The detector–illuminator distance was set at 35 mm. This allowed to have eight recording channels (right hemisphere: Ch 1, Ch 2, Ch, 3, Ch 4; left hemisphere: Ch 5, Ch 6, Ch 7, Ch, 8; Fig. 1). The bundles were assembled into a probe holder that kept the position of the ten optodes fixed. The probe holder was placed over the head to include the underlying PFC with the two photodiodes receivers aligned on Fp1 and Fp2 locations according to the international 10–20 system for the electroencephalography electrode placement. The probe holder provided a stable contact with the scalp for all the optodes. However, optical contact was monitored continuously during the protocol.

### Procedure

Prior to testing the participants received specific instructions according to the role they had been assigned to.

The Examiners were instructed on how administering the CBT: reading the instruction sheet, reproducing the sequences of increasing length as reported on the worksheet, at a speed of one cube per second, and recording the sequences produced by the Examinees on the worksheet.

The Examinees were required to hear and follow the instructions provided by the Examiners, to observe and then to tap the cubes of each sequence in the presentation order.

Both the Examiners and the Examinees were told to avoid speaking during the experiment, after the instruction phase, to avoid any bias in fNIRS recordings. Moreover, as motion artifacts can represent a significant source of noise in fNIRS measures (Pinti et al. 2018a), in our experimental set-up, participants were asked to sit on a chair and not to move their head and body.

After completing fNIRS montage, the dyads of participants moved to the experimental room. The testing was performed in a silent room with constant light and temperature. The Examiner–Examinee dyads were seated face to face at the same distance from the wooden board (50 cm) located on a desktop.

To monitor fNIRS recordings and to mark the start of phase 1 (execution of the Examiner and observation of the Examinee) and of phase 2 (execution of the Examinee and observation of the Examiner) of each trial, an experimenter looked at the scene from a lateral position (1-m away from the dyad), without interfering with the ongoing activities. The events were inserted manually by pressing specific keys on the keyboard of the personal computer used to run the fNIRS acquisition and preprocessing software (OxySoft), when the experimenter detected the start and the end of participants’ motor responses (i.e., when participants raised the hand from the table and then went back to the starting point). Moreover, the experimenter visually inspected Examiner’s accuracy in producing the sequences and inserted an error event by pressing a key on the keyboard in cases of failure in presenting the trial correctly. We planned to exclude from analyses the sequences in which the Examiners were wrong.

Moreover, we planned to exclude dyads in which Examinees showed a spatial span lower than 4 or higher than 8, to comply with the experimental purposes (i.e., the possibility to target specific workload levels; see fNIRS preprocessing and data analysis).

### fNIRS preprocessing and data analysis

Following the data collection procedure, the signal quality as well as the absence of movement artifacts were visually inspected. We planned to exclude channels with low signal to noise ratio, low intensity values (Hocke et al. 2018) and the trials in which fNIRS recording showed visible movement-induced spikes (Brigadoi et al. 2014). We opted for this method to deal with movement interference as participants sitting on a chair and asked not to move their head, produce movement artifacts less likely than when they are assessed standing or walking (Pinti et al. 2018a).

fNIRS data were then preprocessed in OxySoft using a band-pass filter with a low cut of frequency set a 0.01 Hz and a high cut off frequency at 0.1 Hz (Brigadoi et al. 2014; Pinti et al. 2019). The band-pass filter preserves the frequency range between a lower and a higher cut-off frequency and is used to remove noise related to signals at specific frequencies associated with the heart rate (~ 1 Hz) and very low frequency (< 0.04 Hz), and to slightly attenuate respiration rates (~ 0.2–0.3 Hz; Brigadoi et al. 2014; Pinti et al. 2019).

O2Hb and HHb signals were averaged within two ROIs reflecting the right (Ch 1–4) and left (Ch 5–8) PFC, as these regions are known to play a role during short-term memory tasks. Channels in each ROI were chosen based on their anatomical position. As our experimental set-up did not allow to reach high spatial resolution, we averaged the values of each channel in the same ROI separately in the two hemispheres and across all participants for the sequences of the same length.

For each dyad, we calculated the Examinee’s span and analyzed fNIRS data at the Span level, Span − 2 (two levels below the Span), Span− 1 (one level below the Span) and at the Span + 1 (one level above the Span) and Span + 2 (two levels above the Span) levels. In analyzing fNIRS data, we also distinguished between the Execution task, in which the participant (either Examiner or Examinee) reproduced the sequences on the wooden board, and the Observation task, in which the participant (either Examiner or Examinee) observed the sequences reproduced by the other. As the participants of each dyad acted in a complementary way, the Observation task of the Examinee was simultaneous to the Execution task of the Examiner, and viceversa.

A repeated measure 5 × 2 × 2 × 2 Analysis of Variance (ANOVA) with Workload (Span − 2 vs. Span − 1 vs. Span vs. Span + 1 vs. Span + 2), Task (Execution vs. Observation), Hemisphere (Left vs. Right) as within-group factors, and Group (Examiner vs. Examinee) as a between-group factor, was performed on the O2Hb mean measures. A second ANOVA with the same factors was performed on HHb measures. O2Hb and HHb measures were calculated as the average within the time window needed by the participants in the dyads to fulfill the execution/observation of a given cube sequence. Though, since O2Hb provides better contrast and higher amplitude as compared to HHb (Tachtsidis and Scholkmann 2016), we report only results on the former in the main text and describe the latter in a supplementary file (Supplementary File 2).

To assess statistically significant trends in the data a polynomial contrasts’ analysis was performed looking for linear or quadratic trends.

Post hoc comparisons were performed by Bonferroni-corrected tests, with level of significance set at *p* < .05.

## Results

The Examinee’s spatial WM span ranged from a minimum of 4 to a maximum of 8 (*M* = 5.77, SD = 1.07).

The time windows during which the fNIRS signal was averaged closely matched the time windows for the completion of the behavioral task, and ranged 3.21–6.72 s (*M* = 4.36; SD = 0.71) for Span − 2 trials, 3.91–7.12 s (*M* = 5.25; SD = 0.81) for Span − 1 trials, 4.15–8.26 s (*M* = 5.72; SD = 0.83) for Span trials, 5.32–8.88 (*M* = 7.83; SD = 0.63) for Span + 1 trials, and 6.31–9.85 (*M* = 7.83; SD = 0.83) for Span + 2 trials.

No Examiners’ error in sequence administration was observed. Moreover, no channel presented with low signal to noise ratio, or visible spikes induced by movement. Consequently, no data were excluded on these bases. fNIRS data for all task conditions in Examiners and Examinees are reported in Supplementary File 1.

**Fig. 2:**
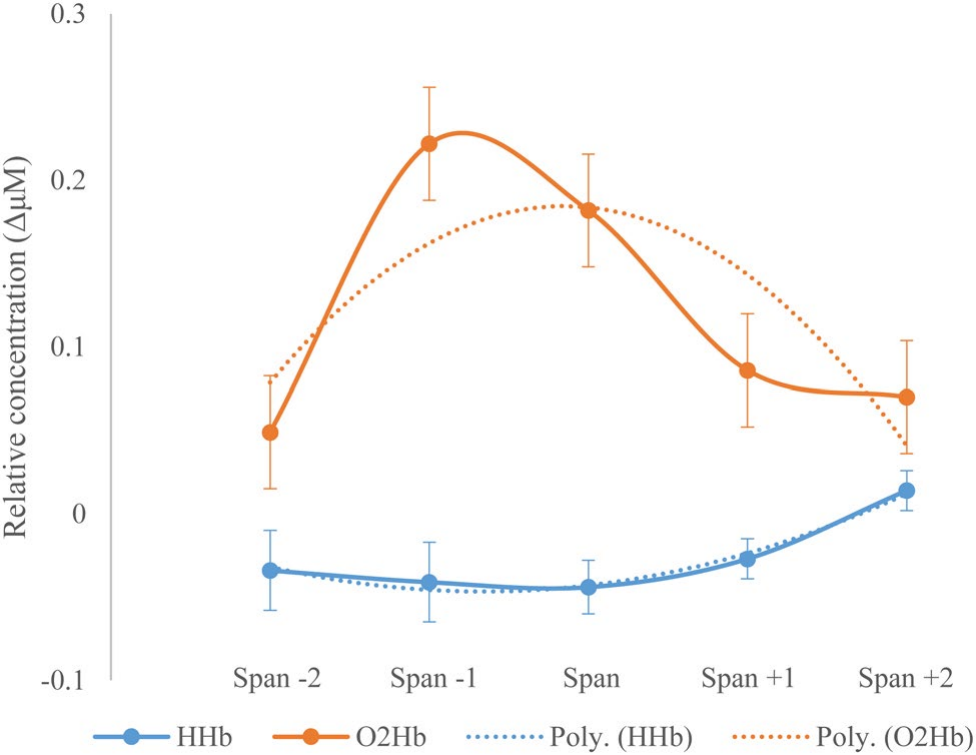
Levels of oxygenated hemoglobin (O2Hb) and deoxygenated hemoglobin (HHb) expressed in ΔμM as a function of cognitive workload (i.e., sequences of increasing length from Examinees’ Span − 2 to Span + 2). Data across Examiner and Examinee and during execution and observation of cube sequences have been collapsed. *Poly.=* theoretical polynomial curve

Results from the ANOVA on the O2Hb mean measures revealed a significant main effect of Workload [*F*(4, 232) = 2.76, *p* = .03, *η*^*2*^*p* = 0.04]. The contrast analysis for this factor revealed a significant quadratic trend [*F*(1, 58) = 6.39, *p* = .01, *η*^*2*^*p* = 0.09], indicating that O2Hb levels tended to increase when the workload approximated (Span − 2: *M* = 0.05, SE = 0.06; Span − 1: *M* = 0.22, SE = 0.07) and reached examinees’ span level (Span: *M* = 0.18, SE = 0.04), and decreased thereafter (Span + 1: *M* = 0.09, SE = 0.03; Span + 2: *M* = 0.07, SE = 0.04; see Fig. 2).

The analysis also demonstrated a significant main effect of Task [*F*(1, 58) = 27.38, *p* < 0.001, *η*^*2*^*p* = 0.32], as O2Hb levels were higher during Execution than Observation (*M* = 0.21, SE = 0.03; *M* = 0.04, SE = 0.03; *p* < .001), and a significant main effect of Group [*F*(1, 58) = 9.21, *p* = .04, *η*^*2*^*p* = 0.14], as O2Hb levels were higher in Examiners as compared to Examinees (*M* = 0.21, SE = 0.04; *M* = 0.04, SE = 0.04; *p* < .01).

A significant Task X Group interaction [*F*(1, 58) = 7.64, *p* < .01, *η*^*2*^*p* = 0.12] demonstrated that O2Hb levels during Execution were higher in Examiners as compared to Examinees (*M* = 0.34, SE = 0.05; *M* = 0.08, SE = 0.05; *p* < .001), and that in Examinees activation was higher during Execution as compared to Observation (*M* = 0.08, SE = 0.04; *p* < .001).

**Fig. 3:**
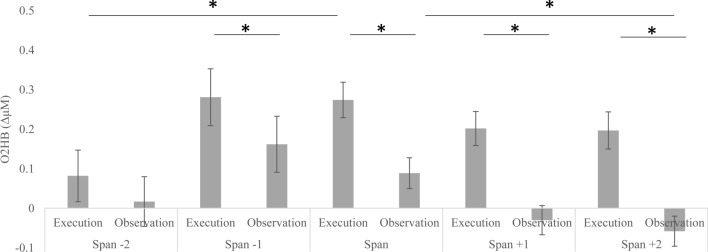
Levels of oxygenated hemoglobin (O2Hb; expressed in ΔμM) during execution and observation of cube sequences as a function of cognitive workload (i.e., sequences of increasing length from Examinees’ Span − 2 to Span + 2). Data across Examiner and Examinee collapsed. *significant at *p* < .05

A *Workload X Task* interaction was also found [*F*(4, 232) = 6.66, *p* < .001, *η*^*2*^*p* = 0.1] as O2Hb levels during Execution were higher at the Span as compared to Span − 2 (*M* = 0.27, SE = 0.04; *M* = 0.08, SE = 0.07; *p* = .03) and during Observation at the Span as compared to the Span + 2 (*M* = 0.89, SE = 0.04; *M* = − 0.06, SE = 0.04; *p* = .04; Fig. 3). In the Span − 1, Span, Span + 1 an Span + 2 higher O2Hb levels were observed during Execution (Span − 1: *M* = 0.28, SE = 0.07; Span: *M* = 0.27, SE = 0.04; Span + 1: *M* = 0.20, SE = 0.04; Span + 2: *M* = 0.19, SE = 0.04) as compared to Observation (Span − 1: *M* = 0.16, SE = 0.07; Span: *M* = 0.09, SE = 0.04; Span + 1: *M* = − 0.03, SE = 0.04; Span + 2: *M* = − 0.06, SE = 0.04; all *p* < .01; Fig. 3).

**Fig. 4:**
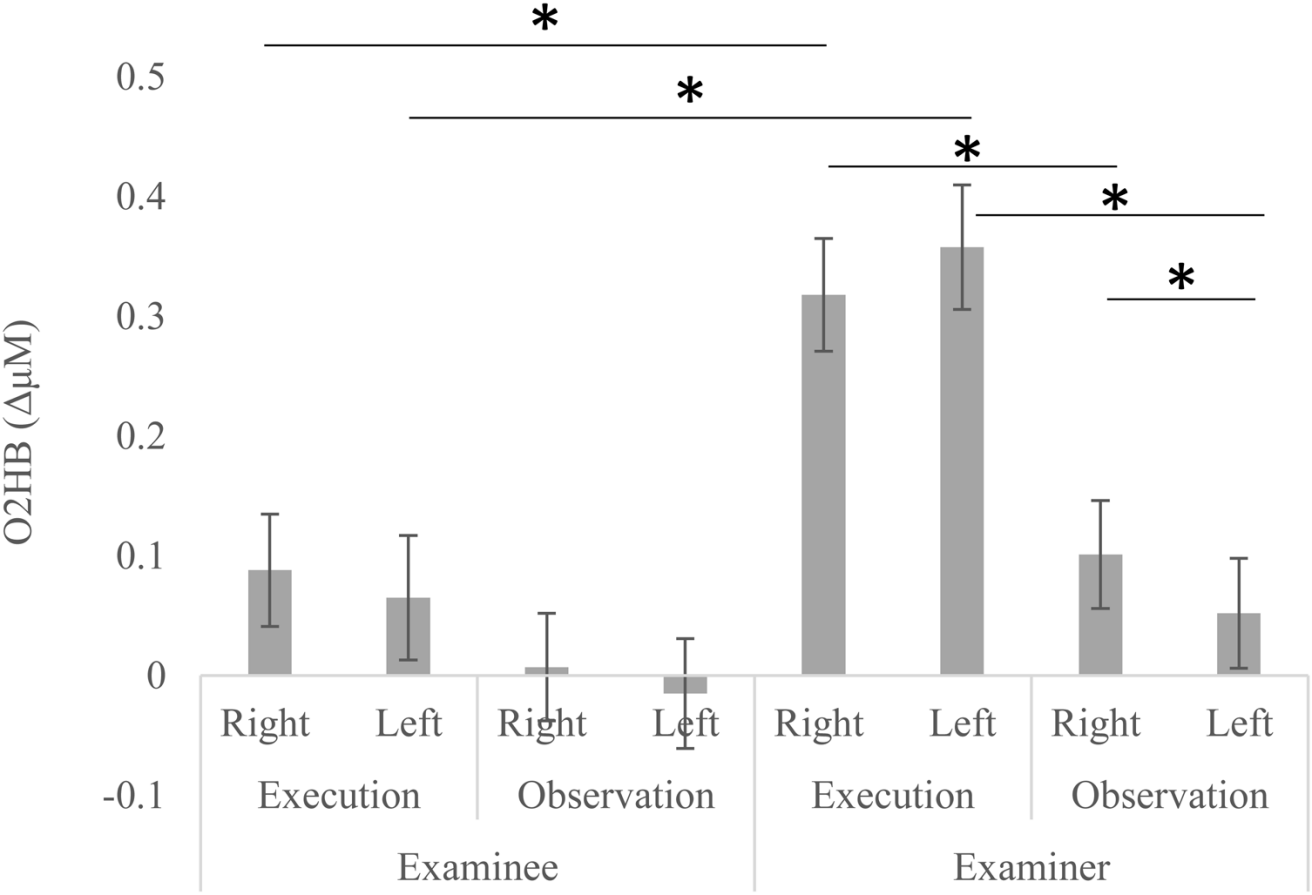
Levels of oxygenated hemoglobin (O2Hb; expressed in ΔμM) in righ- and left-located channels during execution and observation of cube sequences from the Examiner–Examinee dyads. *Significant at *p* < .05

Moreover, the *Task x Hemisphere* [*F*(1, 58) = 7.77, *p* < .01, *η*^*2*^*p* = 0.12] and the *Task X Hemisphere X Group* [*F*(1, 58) = 7.56, *p* < .01, *η*^*2*^*p* = 0.11] interactions were also significant. Indeed, in both the right- and left-located optodes the activation was higher during Execution as compared to Observation (Right: *M* = 0.2, SE = 0.03; *M* = 0.05, SE = 0.03; *p* < .001; Left: *M* = 0.2, SE = 0.04; *M* = 0.02, SE = 0.03, *p* < .001), and during Observation O2Hb levels were higher in the right-located optodes than in left-located optodes (*p* = .01). Crucially, during Execution O2Hb levels were higher in Examiners as compared to Examinees for both the right- and left-located optodes (Right: *M* = 0.32, SE = 0.05; *M* = 0.09, SE = 0.05; *p* = .001; Left: *M* = 0.36, SE = 0.05; *M* = 0.06, SE = 0.05; *p* < .001; Fig. 4). Moreover, in Examiners O2Hb levels were higher during Execution as compared to Observation for both the right- (*M* = 0.32, SE = 0.05; *M* = 0.1, SE = 0.04; *p* < .001) and left-located optodes (*M* = 0.36, SE = 0.05; *M* = 0.05, SE = 0.05; *p* < .001; Fig. 4). Finally, during Observation in Examiners O2Hb levels were higher in the right- as compared to left-located optodes (Fig. 4).

The main effects of Hemisphere [*F*(1, 58) = 1.18, *p* = .28, *η*^*2*^*p* = 0.02], and the interactions between Hemisphere x Group [*F*(1, 58) = 0.53, *p* = .47, *η*^*2*^*p* = 0.01], Workload X Group [*F*(4, 232) = 2.1, *p* = .08, *η*^*2*^*p* = 0.04], Workload X Hemisphere [*F*(4, 232) = 0.36, *p* = .43, *η*^*2*^*p* = 0.01], Workload X Task X Group [*F*(4, 232) = 0.35, *p* = .85, *η*^*2*^*p* = 0.01], Workload X Hemisphere X Group [*F*(4, 232) = 0.86, *p* = .49, *η*^*2*^*p* < 0.01], Workload X Task X Hemisphere [*F*(4, 232) = 0.87, *p* = .48, *η*^*2*^*p* = 0.01] and Workload X Task X Hemisphere X Group [*F*(4, 232) = 0.73 *p* = .57, *η*^*2*^*p* = 0.01] were not significant.

Overall, the results of the ANOVA on HHb levels (see Supplementary File 2) showed that an increase in O2Hb substantially corresponded to a decrease in HHb levels, compatible with the functional hemodynamic response to neuronal activation (Tachtsidis and Scholkmann 2016).

Crucially, the significant quadratic trend of the factor Workload confirmed that as the workload increased, HHb levels decreased, and then increased again, symmetrically to O2Hb levels (Fig. 2).

## Discussion

The aim of the present research was to investigate the involvement of the PFC during CBT administration in an ecological setting characterized by a real interaction within Examiner–Examinee dyads. This was achieved by measuring brain activity with fNIRS devices while young healthy individuals, randomly assigned to the role of Examiners or Examinees, performed the CBT.

The results showed that Examinees’ PFC activity was related to the workload, as it increased as visuo-spatial load increased, and dropped when the workload exceeded their WM Span. Interestingly, the same pattern of brain activation was observed in the Examiners as well, meaning that Examiners’ brain activity increased and decreased in a similar fashion as the Examinees’ one. Within a dyad, O2Hb levels were higher during the execution of the sequences on the CBT board, as compared to when participants had to observe the sequence executed by the other in the same dyad. Another major finding consisted in a higher left-hemisphere activity in the Examiners as compared to the Examinees, particularly during the execution of the CBT sequences.

Taken together, the present results are in line with previous investigations showing the involvement of left and right PFC during the completion of the CBT (Bor et al. 2006; Toepper et al. 2010; Lancia et al. 2018). The present results also demonstrated that PFC activity was modulated by WM load, in line with studies using the N-back task (Owen et al. 2005; Nagel et al. 2009; Fishburn et al. 2014; Herff et al. 2014; Mandrick et al. 2016). However, all the previous studies investigated the neural correlates of increased workload without exceeding participants’ cognitive resources. At variance, in the present experiment, we also measured brain activity when the cognitive workload exceeded participants’ resources, and found a decrease in the level of brain activity in these trials. These results showed a significant quadratic trend between task difficulty and brain activity. This novel finding might suggest that brain activity grew until the cognitive load approached the maximum visuo-spatial processing resources; when the workload exceeded the available visuo-spatial processing resources the participants likely disengaged from the task and did not strongly rely on WM-related brain region. Whether these results were specifically related to a ‘shift’ in participants’ cognitive strategy remains to be elucidated by specifically designed studies.

The parallel pattern of brain activation in Examiner–Examinee dyads during WM tasks has not been reported before. When the task was correctly performed by the Examinees their brain activity increased, and Examiners’ brain activity increased as well. Conversely, when Examinees produced spatial movements not matching the given sequences, such as higher load conditions producing more errors in general, their brain activity decreased, and Examiners’ brain activity decreased as well. Independently from the exact cognitive processes put in motion, these data are in line with recent studies on brain synchronization (Cui et al. 2012; Dommer et al. 2012; Jiang et al. 2012; Liu et al. 2012, 2015, 2016; Baker et al. 2016; Nguyen et al. 2020), showing larger cortical hemodynamic responses during cooperative or conjoint activities, and on functional and neural similarities between action and perception (Meltzoff and Decety 2003; Kokal et al. 2009). In the field of neuropsychological testing, these results are highly relevant, as they seem to suggest a complex interaction between the Examiner and the Examinee during clinical assessment. To date, a few studies have investigated the impact of several features related to the Examiner, the Examinee and the evaluation setting (such as Examiner’s attention/inattention, Examinee’s trait anxiety, the presence of a third-part observer) on the performance on neuropsychological tests (Yantz and McCaffrey 2007; Horwitz and McCaffrey 2008). To what extent the Examiner is able to affect the cognitive performance of the Examinee and which mechanisms are involved in this process have to be investigated in further studies. These studies should also consider the Examiners’ span, in addition to the Examinees’ one, and provide an independent measure of the Examinee’s span performed by a skilled neuropsychologist. Indeed, if one posits that by accident Examiners and Examinees in each dyad had exactly the same span this could account for the similar pattern of neural responses in the participants of the two groups. However, in the present study, the variability in the Examinees’ span (ranging 4–8) made this interpretation quite unlikely.

The lack of relevant left prefrontal activation during observation in our experiment seems to be consistent with hypotheses about the neural bases of WM model (Baddeley, 1992). It is well established that verbal working memory tasks primarily activate left hemispheric brain areas, whereas visuospatial working memory tasks mainly determine right hemispheric activation (e.g.,Clark et al. 2001; Smith et al. 1996; Paulesu et al. 1993; Barbey et al. 2013; Funahashi 2017; Chai et al. 2018). Central executive processes are considered to be related to frontal lobe functions bilaterally involving also parietal regions and constituting a distributed fronto-parietal network (Collette and Van der Linden 2002; Collette et al. 1999; Li et al. 2004; Osaka et al. 2004). Finally, the finding of higher brain activity in the Examiners as compared to the Examinees in the execution task could suggest that the Examiners and the Examinees used different cognitive strategies. Indeed, the Examinees during the execution task had to reproduce observed cube sequences relying on their spatial WM resources, whereas the Examiners likely resorted both to their visuospatial memory (to identify the cube to be tapped) and to their verbal memory (to keep the sequence of cube numbers in their mind). This interpretative framework could be further addressed by comparing PFC activity lateralization in Examiner–Examinee dyads during the administration of verbal and visuospatial WM tests.

However, it has to be acknowledged that in our experimental set-up, the Examiners engaged in additional tasks as compared to the Examinees, and this may have affected PFC activity as well. Indeed, the Examiners were asked to administer an unfamiliar complex task and to record the sequences reproduced by the Examinees. This additional load, which seemed to require multi-tasking and higher cognitive load, could have affected Examiners’ brain activity (Burgess 2000; Stuss and Alexander 2000). However, this effect, if relevant, would have been found during all phases of the experimental procedure regardless of the level of workload as the multi-tasking component in the Examiners was present during the entire experimental task.

Some limitations of the present study have to be acknowledged. The low spatial resolution of the fNIRS and the lack of neuronavigation systems for optode placement precluded strict anatomical inferences on activated brain regions. However, since our aim was to explore for the first time brain activation in Examiner–Examinee dyads during increasing workload in a real setting, we did not focus on narrow brain areas. Moreover, as our main purpose was to describe the levels of brain activity as a function of different workload conditions in the Examiner–Examinee dyads, the experimental procedure did not include a baseline measure in which participants only reproduced or observed some sequences with no concomitant WM load.

In the present study, we enrolled participants within a narrow age range (young master students) as brain activity associated to WM tasks has been demonstrated to be influenced by age (Nagel et al. 2009). Assessing whether individuals’ cognitive reserve could modulate this relationship (Zarantonello et al. 2020) could be a further area of investigation. Moreover, as in the present study the participants had no previous experience with the CBT, and had no opportunity to practice the test before the experimental procedure, it could be worth investigating whether task-induced activity could change with levels of expertise in CBT administration (Causse et al. 2019), so to comprehend whether a reduced cognitive load in managing the task could be associated with lower brain activity levels.

Another significant limitation in fNIRS studies is the scarcity of approaches reliably accounting for systemic interferences. Tachtsidis and Scholkmann (2016) highlighted the possibility of measuring inadvertently fNIRS hemodynamic responses unrelated with neurovascular coupling, such as those arising in the extracerebral layers (i.e., skin blood flow changes), or variations in breath and arousal, and in autonomic nervous system activity. Moreover, Scholkmann et al. (2014a) showed that even inner speech is able to affect cerebral hemodynamics and oxygenation in the anterior PFC due to alterations in the arterial carbon dioxide pressure. In our setting, this might have happened in the Examiners who tried to keep in mind the verbal counterpart of cube sequences. Future studies should overcome these limitations by providing concurrent measurements of physiological parameters during fNIRS recording, and by directly comparing induced brain activity while the Examiners and the Examinees are instructed to use specific strategies while fulfilling the task.

## Conclusions

The present study represents the first demonstration of PFC involvement in CBT executed in an ecological setting in a condition of interaction. Moreover, for the first time, we described here the PFC activation pattern in the Examiner, in addition to that recorded in the Examinee. The finding of symmetric patterns of brain activation in the Examiner–Examinee dyads could suggest a mechanism of brain resonance (Meltzoff and Decety 2003) and confirm similar functional correlates in the action/perception domains, but requires further studies to be fully understood. The use of hyperscanning, a neuroimaging technique allowing the simultaneous recording of the hemodynamic or neuroelectric activities from multiple subjects (Dumas et al. 2011), could help clarifying this point.

We feel that the present findings can open new areas of research paving the way for a deeper understanding of the dynamics occurring during neuropsychological evaluation, which might be quite far from a neutral process as it is usually maintained, and contributing to the new frontiers in cognitive neurosciences (Matusz et al. 2018). Similarly, our results highlighted the potential of simultaneously assessing brain hemodynamic responses in jointly acting individuals in various other research fields where interpersonal performance is involved, such as physician–patient relationship in healthcare (Hardy 2017), vendor-consumer exchange in neuro-marketing (Krampe et al. 2018), or teacher-student communication in educational neurosciences (Bevilacqua et al. 2018).

## Supplementary Information

Below is the link to the electronic supplementary material.

Supplementary file1 (DOCX 33 KB)

Supplementary file2 (DOCX 226 KB)

Supplementary file3 (TIF 647 KB)

Supplementary file4 (TIF 506 KB)

## Data Availability

The data that support the findings of this study are available from the corresponding author, FP, upon reasonable request.
