# Supplementary material for: Brain hemodynamic response in Examiner–Examinee dyads during spatial short-term memory task: an fNIRS study
Source: Exp Brain Res. 2021 Mar 22;239(5):1607–16. doi: 10.1007/s00221-021-06073-0 (PMC8144143; doi:10.1007/s00221-021-06073-0)
Supplement: Supplementary file 1 — Supplementary file1 (DOCX 33 KB) [file 221_2021_6073_MOESM1_ESM.docx]

**Supplementary File 1**

|  |  |  | **Span 2** | | **Span 3** | | **Span 4** | | **Span 5** | | **Span 6** | | **Span 7** | | | **Span 8** | | | **Span 9** | | | **Span 10** | |
| --- | --- | --- | --- | --- | --- | --- | --- | --- | --- | --- | --- | --- | --- | --- | --- | --- | --- | --- | --- | --- | --- | --- | --- |
|  |  |  | HHb | O2Hb | HHb | O2Hb | HHb | O2Hb | HHb | O2Hb | HHb | O2Hb | HHb | O2Hb | HHb | | O2Hb | HHb | | O2Hb | HHb | | O2Hb |
|  |  |  | M  (SE) | M  (SE) | M  (SE) | M  (SE) | M  (SE) | M  (SE) | M  (SE) | M (SE) | M (SE) | M (SE) | M (SE) | M (SE) | M (SE) | | M (SE) | M (SE) | | M (SE) | M (SE) | | M (SE) |
| **Examinee** | Observation | Right | 0.06 | -0.04 | 0.02 | -0.03 | -0.02 | 0.00 | -0.05 | 0.09 | -0.07 | 0.02 | -0.05 | -0.01 | -0.02 | | 0.02 | -0.05 | | -0.10 | -0.01 | | -0.04 |
|  |  |  | (0.15) | (0.36) | (0.13) | (0.26) | (0.09) | (0.28) | (0.10) | (0.28) | (0.10) | (0.27) | (0.11) | (0.26) | (0.08) | | (0.20) | (0.09) | | (0.19) | (0.10) | | (0.29) |
|  |  | Left | 0.05 | -0.05 | 0.03 | -0.08 | 0.00 | -0.01 | -0.04 | 0.07 | -0.04 | 0.00 | -0.04 | -0.01 | -0.02 | | -0.05 | -0.01 | | -0.01 | -0.03 | | -0.11 |
|  |  |  | (0.14) | (0.35) | (0.10) | (0.24) | (0.10) | (0.22) | (0.10) | (0.27) | (0.11) | (0.23) | (0.08) | (0.20) | (0.08) | | (0.16) | (0.12) | | (0.39) | (0.08) | | (0.29) |
|  | Execution | Right | 0.06 | -0.04 | 0.01 | -0.04 | -0.01 | 0.05 | -0.04 | 0.13 | -0.04 | 0.16 | 0.01 | 0.10 | 0.05 | | 0.19 | 0.02 | | 0.06 | 0.05 | | 0.05 |
|  |  |  | (0.12) | (0.39) | (0.11) | (0.29) | (0.09) | (0.29) | (0.08) | (0.27) | (0.07) | (0.22) | (0.10) | (0.22) | (0.10) | | (0.27) | (0.09) | | (0.19) | (0.10) | | (0.21) |
|  |  | Left | 0.02 | -0.12 | 0.01 | -0.08 | 0.00 | 0.05 | -0.03 | 0.12 | -0.04 | 0.12 | 0.00 | 0.10 | 0.01 | | 0.09 | 0.07 | | 0.10 | 0.06 | | 0.07 |
|  |  |  | (0.13) | (0.27) | (0.09) | (0.28) | (0.10) | (0.24) | (0.11) | (0.24) | (0.09) | (0.23) | (0.09) | (0.24) | (0.08) | | (0.27) | (0.12) | | (0.28) | (0.12) | | (0.22) |
| **Examiner** | Observation | Right | 0.01 | 0.09 | -0.05 | 0.08 | -0.06 | 0.30 | -0.05 | 0.09 | -0.06 | 0.12 | -0.03 | -0.04 | -0.02 | | 0.09 | -0.01 | | -0.03 | 0.01 | | -0.14 |
|  |  |  | (0.15) | (0.45) | (0.13) | (0.34) | (0.10) | (0.39) | (0.08) | (0.28) | (0.07) | (0.15) | (0.07) | (0.17) | (0.06) | | (0.29) | (0.06) | | (0.24) | (0.06) | | (0.17) |
|  |  | Left | 0.00 | 0.09 | -0.03 | 0.10 | -0.09 | 0.23 | -0.08 | 0.03 | -0.06 | 0.05 | -0.05 | -0.13 | -0.04 | | 0.05 | 0.01 | | -0.07 | 0.01 | | -0.13 |
|  |  |  | (0.17) | (0.45) | (0.14) | (0.38) | (0.10) | (0.37) | (0.09) | (0.29) | (0.08) | (0.21) | (0.07) | (0.17) | (0.07) | | (0.33) | (0.07) | | (0.24) | (0.05) | | (0.17) |
|  | Execution | Right | 0.03 | 0.13 | -0.03 | 0.04 | -0.02 | 0.40 | -0.02 | 0.30 | -0.02 | 0.39 | 0.02 | 0.30 | 0.00 | | 0.26 | 0.04 | | 0.15 | 0.04 | | 0.04 |
|  |  |  | (0.14) | (0.45) | (0.14) | (0.37) | (0.12) | (0.49) | (0.07) | (0.24) | (0.08) | (0.21) | (0.07) | (0.38) | (0.09) | | (0.34) | (0.06) | | (0.21) | (0.08) | | (0.24) |
|  |  | Left | 0.02 | 0.15 | 0.01 | 0.10 | -0.02 | 0.40 | -0.02 | 0.35 | 0.01 | 0.45 | 0.04 | 0.32 | 0.02 | | 0.31 | 0.07 | | 0.25 | 0.08 | | 0.15 |
|  |  |  | (0.16) | (0.43) | (0.15) | (0.40) | (0.12) | (0.45) | (0.08) | (0.23) | (0.08) | (0.29) | (0.07) | (0.36) | (0.08) | | (0.42) | (0.07) | | (0.26) | (0.09) | | (0.22) |

**Table 1.** Means (M) and Standard Errors (SE) for deoxygenated (HHb) and oxygenated (O2Hb) hemoglobin during the Corsi Block Tapping Test (from Span 2 to Span 10) in Examinee and Examiner.

**Table 2**. Means (M) and Standard Errors (SE) for deoxygenated (HHb) and oxygenated (O2Hb) hemoglobin for the workload levels (from Span -2 to Span +2) in Examinee and Examiner

|  |  |  | **Span -2** | | **Span -1** | | **Span** | | **Span +1** | | **Span +2** | |
| --- | --- | --- | --- | --- | --- | --- | --- | --- | --- | --- | --- | --- |
|  |  |  | HHb | O2Hb | HHb | O2Hb | HHb | O2Hb | HHb | O2Hb | HHb | O2Hb |
|  |  |  | M  (SE) | M  (SE) | M  (SE) | M  (SE) | M  (SE) | M  (SE) | M  (SE) | M  (SE) | M  (SE) | M  (SE) |
| **Examinee** | Observation | Right | -0.02 | -0.03 | -0.01 | 0.02 | -0.07 | 0.10 | -0.06 | -0.04 | -0.01 | -0.01 |
|  |  |  | (0.12) | (0.23) | (0.11) | (0.23) | (0.09) | (0.25) | (0.08) | (0.25) | (0.09) | (0.23) |
|  |  | Left | 0.01 | -0.04 | -0.01 | 0.01 | -0.05 | 0.04 | -0.04 | -0.09 | -0.01 | 0.00 |
|  |  |  | (0.10) | (0.22) | (0.10) | (0.22) | (0.10) | (0.25) | (0.10) | (0.21) | (0.09) | (0.26) |
|  | Execution | Right | -0.03 | -0.03 | -0.03 | 0.04 | -0.05 | 0.19 | 0.02 | 0.08 | 0.06 | 0.15 |
|  |  |  | (0.10) | (0.24) | (0.08) | (0.27) | (0.08) | (0.25) | (0.09) | (0.22) | (0.09) | (0.22) |
|  |  | Left | -0.02 | -0.04 | -0.03 | 0.06 | -0.03 | 0.13 | 0.02 | 0.08 | 0.06 | 0.13 |
|  |  |  | (0.09) | (0.21) | (0.08) | (0.29) | (0.10) | (0.24) | (0.09) | (0.22) | (0.11) | (0.25) |
| **Examiner** | Observation | Right | -0.08 | 0.08 | -0.08 | 0.35 | -0.07 | 0.13 | -0.05 | 0.03 | -0.03 | -0.08 |
|  |  |  | (0.12) | (0.36) | (0.10) | (0.38) | (0.10) | (0.22) | (0.07) | (0.18) | (0.06) | (0.23) |
|  |  | Left | -0.09 | 0.06 | -0.09 | 0.27 | -0.07 | 0.09 | -0.06 | -0.02 | -0.03 | -0.14 |
|  |  |  | (0.15) | (0.40) | (0.12) | (0.39) | (0.10) | (0.27) | (0.09) | (0.25) | (0.06) | (0.21) |
|  | Execution | Right | -0.03 | 0.17 | -0.04 | 0.50 | -0.02 | 0.37 | -0.01 | 0.30 | 0.02 | 0.24 |
|  |  |  | (0.11) | (0.39) | (0.12) | (0.41) | (0.10) | (0.28) | (0.10) | (0.25) | (0.06) | (0.31) |
|  |  | Left | -0.02 | 0.22 | -0.03 | 0.51 | 0.00 | 0.41 | 0.01 | 0.38 | 0.05 | 0.27 |
|  |  |  | (0.13) | (0.42) | (0.13) | (0.41) | (0.10) | (0.29) | (0.08) | (0.33) | (0.06) | (0.31) |
