# Supplementary material for: Brain hemodynamic response in Examiner–Examinee dyads during spatial short-term memory task: an fNIRS study
Source: Exp Brain Res. 2021 Mar 22;239(5):1607–16. doi: 10.1007/s00221-021-06073-0 (PMC8144143; doi:10.1007/s00221-021-06073-0)
Supplement: Supplementary file 2 — Supplementary file2 (DOCX 226 KB) [file 221_2021_6073_MOESM2_ESM.docx]

**Supplementary File 2**. Results of statistical analyses on deoxygenated hemoglobin

Results from the ANOVA on the HHb mean measures revealed a tendency to significance of Workload [F(4, 232)= 2.31, p=.06, η²p= .04] and a significant main effect of Task [F(1, 58)= 19.84, p< .001, η²p= .25], as HHb levels were higher during Execution as compared to Observation (M= -.01, SE= .01; M= -.05, SE= .01; p< .001). There was a significant quadratic trend of the factor Workload [F(1, 58)= 2.48, p= .04, *η²p*= .07], indicating that as the workload increased, HHb levels decreased, and then increased again (Figure 2 of the main text).

A significant Task X Group interaction [F(1, 58)= 4.12, p= .05, η²p= .06] demonstrated that in the Examiners HHb levels during Execution were higher as compared to Observation (M= -.01, SE= .02; M= -.06, SE= .02; p< .001). A Workload X Task interaction was also found [F(4, 232)= 5.88, p< .001, η²p= .09] as HHb levels during Execution were higher at Span+2 (M= .05, SE= .01) as compared to those at Span-1 and Span (M= -.03, SE= .02; M= -.02, SE= .02; all p< .01; Supplementary Figure 1). Moreover, at Span -2, Span, Span +1 and Span +2, HHb levels were higher during Execution as compared to Observation (Span -2: M= -.02, SE= .02; M= -.04, SE= .03; p= .04; Span: M= -.02, SE= .02; M= -.07, SE= .23; p< .001; Span +1: M= .001, SE= .01; M= -.05, SE= .01; p< .001; Span +2: M= .05, SE= .01; M= -.02, SE= .01; p< .04; Supplementary Figure 1).


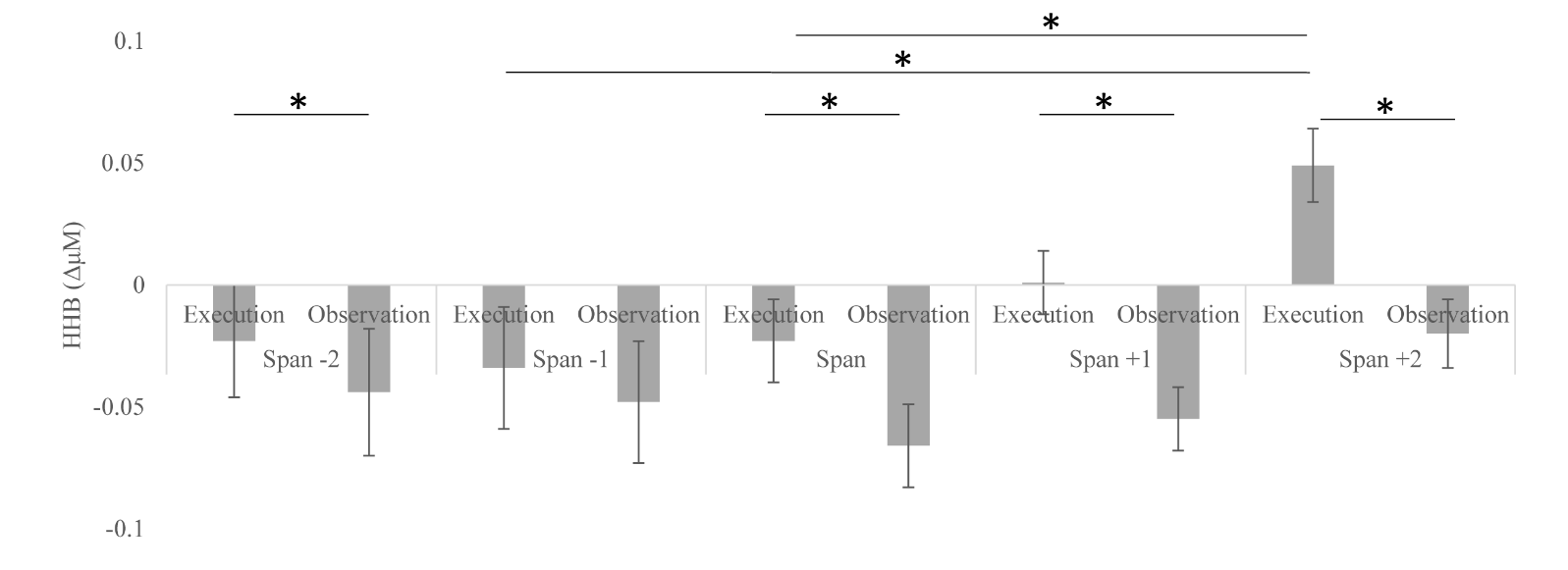


**Supplementary Figure 1.** Levels of deoxygenated hemoglobin (HHb; expressed in ΔμM) during execution and observation of cube sequences in the several workload conditions. Data across Examiner and Examinee collapsed; *significant at p< .05

A Task X Workload X Group [F(4, 232)= 4.03, p< .01, η²p= .06] and a Task X Hemisphere X Group [F(1, 58)= 9.24, p< .01, η²p= .14] interactions were found. Indeed, in Examiners there were higher HHB levels during Execution as compared to Observation during all the workload levels (Span-2: M= -.02, SE= .03; M= -.08; SE= .04; Span-1: M= -.04, SE= .04; M= -.09; SE= .03; Span: M= -.01, SE= .02; M= -.07; SE= .02; Span+1: M= -.004, SE= .02; M= -.06; SE= .02; Span+2: M= .04, SE= .02; M= -.03; SE= .02; all p< .05), whereas in Examinees HHB levels were higher during Execution as compared to Observation only at Span +1 (M= .01, SE= .02; M= -.05, SE= .02, p< .01) and Span +2 (M= .06, SE= .02; M= -.01, SE= .2; p= .001); in Examinees there were higher HHB levels during Execution at Span +2 (M= .06, SE= .02) as compared to Span (M= -.04, SE= .02; p=.05). Moreover, while in Examiners for both the right and left hemispheres there where higher levels of HHB during Execution (Right: M= -.01; SE= .02; Left: M= .001, SE= .02) as compared to Observation (Right: M= -.06, SE= .02; Left: M= -.07, SE= .02; all p< .01), in Examinees this happened only in the right hemisphere (M= -.01, SE= .02; M= -.03, SE= .02; p= .05; Supplementary Figure 2).


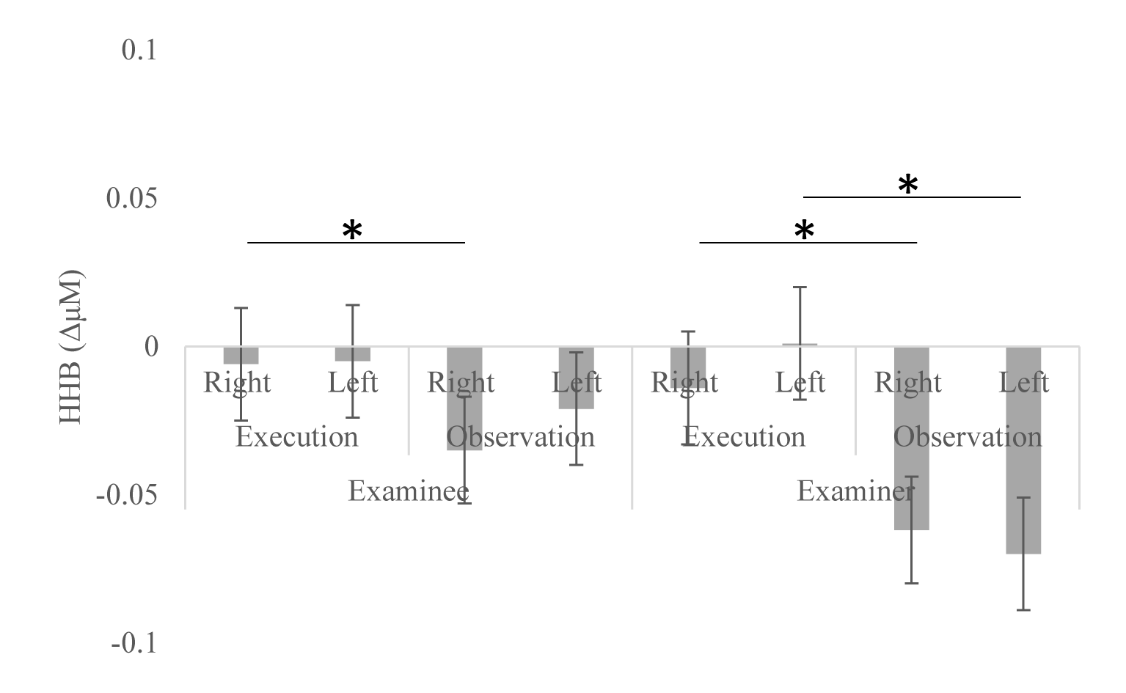


**Supplementary Figure 2.** Levels of deoxygenated hemoglobin (HHb; expressed in ΔμM) in righ- and left-located channels during execution and observation of cube sequences from the Examiner-Examinee dyads. *significant at p< .05

The main effects of Group [F(1, 58)= .63, p= .43, η²p= .01], Hemisphere [F(1, 58)= 1.34, p= .25, η²p= .02], the interactions between Hemisphere x Group [F(1, 58)= .12, p= .73, η²p= .002], Workload X Hemisphere [F(4, 232)= .43, p= .78, η²p= .01], Task X Hemisphere [F(1, 58)= .6, p= .44, η²p= .01], Workload X Hemisphere X Group [F(4, 232)= .46 , p= .76, η²p= .01], Workload X Task X Hemisphere [F(4, 232)= 1.18, p= .32, η²p= .02] and Workload X Task X Hemisphere X Group [F(4, 232)= 1.95 p= .1, η²p= .03] were not significant.
