# Supplementary figures and images for: Brain hemodynamic response in Examiner–Examinee dyads during spatial short-term memory task: an fNIRS study
Source: Exp Brain Res. 2021 Mar 22;239(5):1607–16. doi: 10.1007/s00221-021-06073-0 (PMC8144143; doi:10.1007/s00221-021-06073-0)

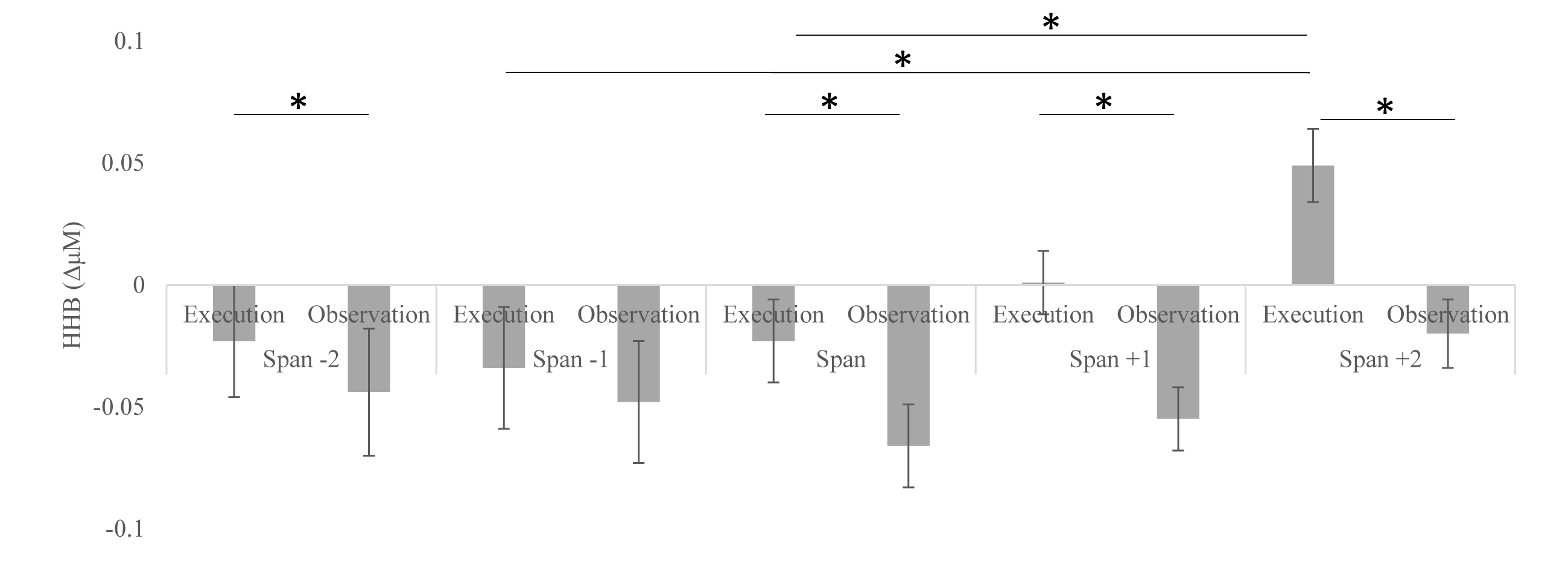

Supplement: Supplementary file 3 — Supplementary file3 (TIF 647 KB) [file 221_2021_6073_MOESM3_ESM.tif]

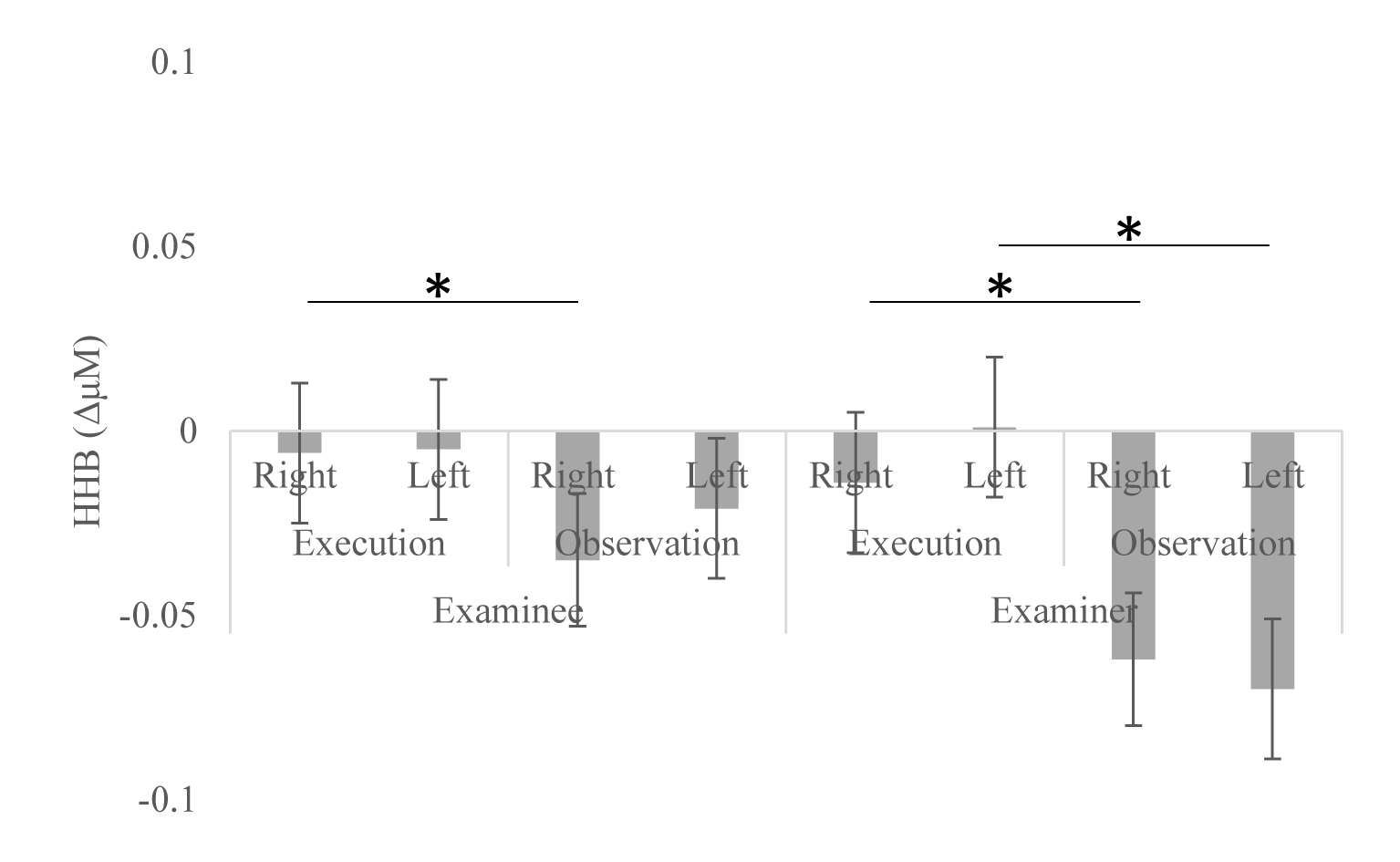

Supplement: Supplementary file 4 — Supplementary file4 (TIF 506 KB) [file 221_2021_6073_MOESM4_ESM.tif]
